# Vegetation–Atmosphere Water Deficit as the Primary Control on Alpine Steppe and Forest Coverage: An Empirical Assessment from the Altay Mountains, Northwestern China

**DOI:** 10.3390/biology15110879

**Published:** 2026-06-02

**Authors:** Qiao Xu, Yan Xu, Dong Cui, Tao Lin, Zhiguo Miao, Yincheng Gong, Aishajiang Aili, Fabiola Bakayisire

**Affiliations:** 1 College of Resources and Environment, Yili Normal University, Yining 835000, China; 2 Institute of Resources and Ecology, Yili Normal University, Yining 835000, China; 3 Desert-Oasis Ecological Monitoring and Restoration Engineering Technology Innovation Center, Ministry of Natural Resources, Urumqi 830002, China; 4 Xinjiang Uygur Autonomous Region Ecological Water Resources Research Center (Academician and Expert Workstation of the Department of Water Resources of the Xinjiang Uygur Autonomous Region), Urumqi 830000, China; 5 Xinjiang Comprehensive Land Consolidation and Rehabilitation Center, Urumqi 830002, China; 6 Research Center of Desert and Oasis Ecology, Xinjiang Institute of Ecology and Geography, Chinese Academy of Sciences, Urumqi 830011, China

**Keywords:** Altay Mountains, drought, extreme climate indices, NDVI, SPEI, TVDI, vegetation–atmosphere water deficit

## Abstract

Mountain vegetation in dryland regions is highly sensitive to climatic variability, particularly changes in water availability and atmospheric demand. This study assessed the relationships between vegetation coverage and climatic factors in the Chinese Altay Mountains from 2000 to 2024 using MODIS NDVI data, meteorological observations, drought indices, and extreme climate indicators. Pixel-based correlation analysis and directional interaction classification were used to evaluate the spatial consistency and divergence between vegetation dynamics and climate variability. The results showed that water availability was the dominant factor controlling vegetation cover. Annual precipitation, SPEI, and precipitation-related extreme indices were generally positively associated with vegetation coverage, whereas warmth-related indices such as GSL, WSDI, and TX90 were mostly negatively associated with vegetation coverage. Temperature showed a spatially variable effect, with warming tending to suppress vegetation in water-limited low- and middle-elevation areas but potentially benefiting vegetation in cold-limited high-elevation zones. SPEI showed a more consistent relationship with vegetation coverage than TVDI, indicating that cumulative climatic water balance better captured regional vegetation drought responses than surface dryness alone. These findings highlight the importance of vegetation–atmosphere water deficit in regulating mountain vegetation dynamics and provide a scientific basis for ecological conservation and water resource management in the Altay Mountains.

## Simple Summary

Mountain plants in dry regions are increasingly affected by climate change, especially by changes in rainfall, heat, and drought. This study examined how plant cover in the Chinese Altay Mountains changed from 2000 to 2024 and how these changes were related to temperature, rainfall, drought, and extreme weather. The aim was to identify which climate factors most strongly influence vegetation growth in this important mountain ecosystem. The results showed that water supply was the main factor controlling plant cover. More rainfall and wetter conditions generally supported vegetation growth, while drought and warm extremes often reduced plant cover. Temperature had mixed effects: warming could help plants in cold, high mountain areas, but it often harmed vegetation in lower and drier areas by increasing water stress. Moderate rainy days were especially beneficial, while longer warm periods and more warm days were mostly unfavorable. These findings show that protecting mountain vegetation requires careful management of water resources and close monitoring of drought and extreme heat. The results can support ecological conservation, forest protection, and climate adaptation planning in the Altay Mountains.

## 1. Introduction

The Altay Mountains span the territories of China, Kazakhstan, Russia, and Mongolia and constitute one of Central Asia’s most important mountain ecosystems [1]. Within China, the Altay Mountains occupy the southern slope of the central mountain section and function as a vital ecological shield in northern Xinjiang. This region contains large areas of forest, grassland, and peatland wetland ecosystems [2,3,4]. Siberian larch (*Larix sibirica* Ledeb.), Siberian spruce (*Picea obovata* Ledeb.), and silver birch (*Betula pendula* Roth), forming diverse forest types that collectively account for 47% of Xinjiang’s natural forest resources [5]. The widespread and well-preserved peat swamp ecosystems in this area further highlight its significance as an important regional carbon reservoir and water conservation zone [6].

The Altay Mountains are dominated by a continental climate, which is shaped by their inland position in Eurasia and their great distance from maritime influences [7]. Clear seasonal differences are evident across the region. Spring is marked by rapid temperature increases and frequent winds, summer is short and relatively cool, autumn brings sunny conditions accompanied by abrupt cooling, and winter is prolonged and extremely cold. Mean annual temperature varies with elevation, remaining below −9 °C in the middle and high mountain zones at 1400–2600 m, while staying below 4 °C in the low mountain and hilly zones [8]. Precipitation also shows a strong altitudinal gradient, rising by approximately 30–80 mm for every 100 m increase in elevation, from about 200–300 mm per year in the low mountain belt to roughly 600–800 mm per year in the high mountain belt. This pronounced spatial heterogeneity in both temperature and precipitation creates diverse ecological niches and makes the region particularly sensitive to climate variability [8,9].

Over the past several decades, global climate change has become increasingly pronounced, and warming in mountain ecosystems has generally occurred more rapidly than in adjacent lowland regions [10,11]. The Altay Mountains have also been affected by these climatic changes, including increasing temperatures, shifts in precipitation patterns, and more frequent extreme weather events. Such changes may seriously affect vegetation condition, forest productivity, and key ecosystem functions, such as water regulation, carbon storage, and biodiversity maintenance [12,13]. Therefore, clarifying how vegetation in the Altay Mountains responds to long-term climate variation and episodic extreme events is essential for formulating effective adaptive management strategies.

Earlier research has shown that vegetation development in arid and semi-arid regions is largely constrained by water availability [14,15]. In cold mountain systems such as the Altay Mountains, however, temperature is also a key limiting factor, especially at higher elevations [16]. The combined effects of temperature and precipitation, operating through evapotranspiration, soil water storage, and physiological tolerance limits of plants, can lead to complex and non-linear vegetation responses that differ among elevation zones and vegetation communities. In addition, extreme climatic events, including heatwaves, persistent drought, and intense precipitation, may influence vegetation more strongly than gradual changes in average climate conditions [17,18,19]. Nevertheless, the responses of vegetation in the Altay Mountains to specific climate-extreme indicators, such as warm day frequency (TX90), warm spell duration index (WSDI), growing season length (GSL), summer days (SU25), maximum daily minimum temperature (TNx), and heavy precipitation metrics including SDII, R95p, and R10, have not yet been sufficiently quantified [3,20,21].

Previous studies in the Altay Mountains have improved understanding of vegetation productivity and evapotranspiration responses to surface meteorological factors, but most have focused on mean climate conditions or single ecological indicators. Studies from other mountain and dryland regions, including the Qinghai–Tibet Plateau, Central Asia, Europe, western North America, and the Andes, have shown that vegetation responses to climate variability are strongly mediated by elevation, water availability, drought legacy effects, and extreme events. However, a spatially explicit framework that jointly evaluates mean climate variables, meteorological drought, surface dryness, and extreme climate indices remains limited for the Chinese Altay Mountains. Therefore, the novelty of this study lies in integrating annual temperature and precipitation, SPEI, TVDI, and eight extreme climate indices with pixel-based correlation analysis and directional interaction classification. This approach allowed us to distinguish coordinated and non-coordinated vegetation–climate responses and to identify whether vegetation dynamics were mainly controlled by mean climate conditions, atmospheric water deficit, or extreme climatic events.

To address this knowledge gap, long-term meteorological observations from seven stations and MODIS MOD13Q1 NDVI data were integrated to assess vegetation–climate relationships across the Chinese Altay Mountains from 2000 to 2024. Pixel-based Pearson correlation analysis and directional interaction classification were applied to quantify the spatial relationships between vegetation coverage and mean climate variables, drought indices, and extreme climate indicators. Particular attention was given to identifying the dominant climatic controls on vegetation dynamics and distinguishing coordinated from non-coordinated vegetation responses.

The results were expected to improve understanding of mountain vegetation responses to climate variability and to provide scientific support for forest conservation, ecological restoration, and water resource management in the Altay Mountains. Given the role of this region as an important ecological barrier in northern Xinjiang, such evidence is essential for strengthening regional ecological security under ongoing climate change.

## 2. Data and Methods

### 2.1. Study Area Description

**Figure 1:**
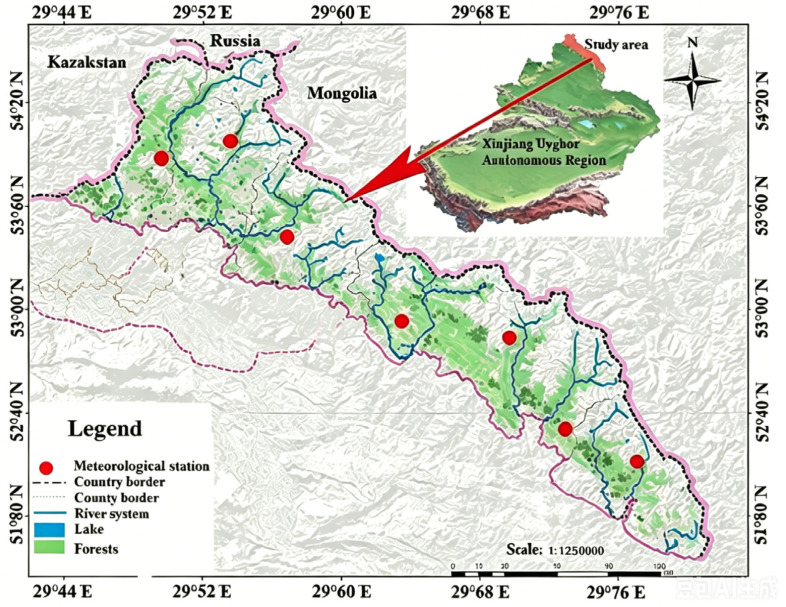
Location and surrounding environment of study area. Note: Driving number of map: GS (2024) 0650) [24]. The pink line in the figure is the counties or district’s border.

The Altay Mountains extend through China, Kazakhstan, Russia, and Mongolia, running for about 2000 km along a northwest–southeast axis [22,23]. The present study concentrates on the Chinese section of the Altay Mountains, situated on the southern slope of the central part of the range. This section stretches approximately 450 km from west to east and 80–150 km from north to south. From northwest to southeast, the mountains gradually become narrower, with the terrain changing from a broad and high-elevation landscape in the northwest to a lower and more confined landform in the southeast (Figure 1). The study area has a total area of approximately 2.6 × 10^4^ km^2^ [8].

The Altay region is characterized by broad expanses of forest, grassland, and wetland ecosystems, which collectively support the region’s key ecological functions [25]. Forests cover approximately 9802 km^2^, representing 37.7% of the area, whereas wetlands occupy about 800 km^2^, or nearly 3% of the total regional area [26,27]. The forest vegetation is mainly dominated by Siberian larch (*Larix sibirica*), accompanied by other important tree species such as Siberian spruce (*Picea obovata*) and silver birch (*Betula pendula*) [28,29]. Together, these species form several major forest communities, including larch forests, spruce forests, mixed larch–spruce forests, and birch-dominated mixed forests [30]. The region also contains numerous peat swamps, which constitute the largest and best-preserved peat swamp system in Xinjiang and represent an important carbon reservoir with substantial water conservation value [31,32].

Owing to its inland position in the Eurasian continent and its long distance from marine influences, the Altay Mountains are governed by a typical continental climate [33]. The region has distinct seasonal characteristics: spring is marked by rapid warming and frequent winds, summer is short and cool, autumn is generally sunny but accompanied by rapid temperature declines, and winter is prolonged and cold. The mean annual temperature is about −2 °C, while the recorded extreme maximum temperature reaches 33.3 °C [8]. Permanent snow cover occurs above elevations of approximately 3100–3300 m [34]. In the middle and high mountain zones, at elevations of 1400–2600 m, the mean annual temperature remains below −9 °C, and even in July, the warmest month, the average temperature is only around 15 °C [35,36]. The annual temperature amplitude is approximately 30 °C, while the daily temperature range is about 12 °C [37]. In the low mountain and hilly zones, the mean annual temperature is generally below 4 °C [38]. Precipitation shows a clear elevation-dependent pattern, increasing by roughly 30–80 mm with every 100 m rise in altitude. Annual precipitation is approximately 200–300 mm in the low mountain belt, 300–500 mm in the middle mountain belt, and 600–800 mm in the high mountain belt, with an overall decreasing trend from north to south and from west to east [39]. Therefore, the region has substantial ecological and water-conservation significance for northern Xinjiang [25]. The Altay Mountains, therefore, represent an important ecological barrier and water-conservation region in northern Xinjiang [25].

### 2.2. Data Sources

Daily meteorological records for 2000–2024 were collected from seven meteorological stations distributed around the Altay Mountains, namely Altay, Burqin, Habahe, Jimunai, Fuhai, Fuyun, and Qinghe. These data were obtained from the China Meteorological Data Sharing Service Network (https://weather.cma.cn/, accessed on 12 October 2025). To improve data reliability and ensure the robustness of subsequent analyses, rigorous quality-control procedures were conducted [40]. Because the selected meteorological stations are relatively well distributed across the study region, the inverse distance weighting (IDW) interpolation method was used to spatially interpolate the station-based observations [41]. Daily average temperature and precipitation were then used to examine the relationships between climatic conditions and variations in vegetation cover.

The NDVI dataset used in this study was derived from the MODIS MOD13Q1 product (sourced from the NASA EOSDIS Land Processes Distributed Active Archive Center [LP DAAC] operated by the U.S. Geological Survey [USGS] Earth Resources Observation and Science [EROS] Center, Sioux Falls, SD, USA), which provides 16-day composite data at a spatial resolution of 250 m. The dataset covered the period from January 2000 to December 2024 [42]. Before analysis, the remote sensing images were preprocessed through format conversion, image mosaicking, projection transformation, and extraction of the study area. To minimize noise and improve the quality of the vegetation signal, the Savitzky–Golay filtering method and maximum value composite (MVC) approach were applied to the NDVI time series, producing annual NDVI datasets that represent optimal vegetation growth conditions [43]. Savitzky–Golay filtering was adopted because it is simple in principle, computationally efficient, and effective for smoothing time-series data [44]. The MVC technique, similar to the procedure used for AVHRR-NDVI datasets, selects the maximum NDVI observation for each pixel within a given period to represent vegetation status during that interval [45]. As a gridded Level-3 product with 16-day temporal resolution and 250 m spatial resolution, MOD13Q1 is widely used for monitoring vegetation dynamics, identifying land-cover changes, and characterizing land-surface biophysical properties, including vegetation coverage and land-cover conversion [42,46].

### 2.3. Definition of Extreme Weather Events

Climate can be understood as the statistical distribution of weather conditions over time [17]. From a statistical perspective, weather events are generally regarded as extreme when they deviate markedly from the long-term average state and occur with relatively low probability [47].

Extreme weather events are commonly classified into two broad types. The first type refers to recurrent weather extremes that may occur in most years, whereas the second type includes rare and severe events that do not occur every year at a given location. Such events are usually characterized by low frequency, unusually high or low intensity, and substantial social or economic impacts [48]. In probabilistic terms, extreme weather and climate events are typically defined as phenomena located at the tails of the probability distribution, often with an occurrence probability of 10% or lower [17]. These events may include intense rainstorms, floods, prolonged droughts, typhoons, snowstorms, extreme cold events, and heatwaves [17].

#### 2.3.1. Extreme Temperature Indicators

Following the standards established by the World Meteorological Organization (WMO) Commission for Climatology, extreme climate indices are defined and calculated using the framework of “Climate Change Detection and Indices” [49]. This approach has been widely adopted by researchers in China and elsewhere for the long-term analysis of extreme climate events [50]. The selected extreme climate indicators were defined according to WMO/ETCCDI climate extreme index criteria and previous applications in climate-change detection studies [49,50]. The definitions, units, and calculation criteria are summarized in Table 1.

#### 2.3.2. Extreme Precipitation Indicators

In this study, Bonsal’s non-parametric method was used to identify the threshold for extreme precipitation. The detailed calculation procedure is described as follows [51]:

Assume that a meteorological variable contains *N* observations. These *N* values are first sorted in ascending order, and the probability that a given value is less than or equal to *x_m_*:

$$P=\left(m-0.31\right)/\left(n+0.38\right)$$

where *m* is the serial number of *x*.

#### 2.3.3. Drought Indicators

(1) The Standardized Precipitation–Evapotranspiration Index (SPEI) is a multi-timescale drought indicator used to evaluate drought severity on the basis of climatic water balance [52]. It is calculated from the difference between precipitation (P) and potential evapotranspiration (PET), which represents atmospheric water demand through evaporation and plant transpiration. In essence, SPEI standardizes the anomaly of the climatic water balance, expressed as precipitation minus potential evapotranspiration, relative to the long-term mean condition. Because the standardized values have a mean of zero and a variance of one, SPEI enables drought conditions to be compared across different regions and time scales [52].
(2) The Temperature Vegetation Dryness Index (TVDI) is a commonly used remote-sensing index for estimating surface soil moisture conditions and detecting agricultural drought [53]. This index is mainly derived from the relationship between land surface temperature (LST) and vegetation indices such as the Normalized Difference Vegetation Index (NDVI). Conceptually, TVDI reflects whether a vegetated surface is warmer than expected under a given vegetation condition. When plants experience water stress, transpiration is reduced, leading to higher canopy or surface temperatures. TVDI captures this thermal response and uses it to indicate the degree of surface dryness [53].

### 2.4. Statistical Methods

#### 2.4.1. Climate Tendency Method

Using the time series of meteorological variables, time (*t*) was treated as the independent variable, whereas each meteorological factor was regarded as the dependent variable [54]. Specifically, *Y* was used to represent a given meteorological variable, and *t* denoted the corresponding time. A linear regression model was then established to quantify the temporal variation in each meteorological factor [54]. In addition, the Pearson correlation coefficient was used to evaluate the linear association between vegetation coverage and meteorological variables [54,55]. The climatic tendency rate was calculated as follows:

Y_i_ = a_0_ + a_1_t_i_ (1)

where Y_i_ denotes the meteorological factor, t_i_ is time, a_1_ is the linear trend term, and a_1_ × 10 gives the climate tendency rate per decade (units: per 10 years). A negative value indicates a decreasing trend in the meteorological factor over time, while a positive value indicates an increasing trend. The larger the absolute value of a_1_, the more pronounced the trend [54].

#### 2.4.2. Correlation Analysis

The dimensionless climate trend coefficient r_xt_ is obtained using the following equation [54]:


(2) $$r_{\mathrm{xt}}=\frac{\sum_{t=1}^{n}\left(x_{t}-x)(t-\frac{n+1}{2}\right)}{\sqrt{\sum_{t=1}^{n}{\left(x_{t}-x\right)}^{2}{\left(t-\frac{n+1}{2}\right)}^{2}}}$$

In this equation, r_xt_ is the trend coefficient, whose significance can be tested using the t-distribution [54,55]. The relationship between the tendency rate (b) and the trend coefficient (r_xt_) is:

(3) $$b=r_{xt}(\sigma_{x}/\sigma_{t})$$

where σ_x_ and σ_t_ are the standard deviations of the factor sequence and the time sequence, respectively.

The Pearson correlation coefficient (R) is used to describe the relationship between vegetation productivity and precipitation/temperature [54,55]. A high R-value indicates a strong correlation, while a low R-value indicates a weaker correlation. Grid-based correlation analyses were performed using custom code written in MATLAB software (version R2024b) [55].

#### 2.4.3. Pixel-Based Correlation and Directional Interaction Classification

Pixel-based Pearson correlation analysis was conducted to quantify the relationship between annual maximum NDVI and each climatic variable, including mean annual temperature, annual precipitation, SPEI, TVDI, and extreme climate indices. Correlation coefficients were classified into eight intervals: <−0.6, −0.6 to −0.4, −0.4 to −0.2, −0.2 to 0, 0 to 0.2, 0.2 to 0.4, 0.4 to 0.6, and >0.6. To further examine coordinated and non-coordinated vegetation–climate relationships, each pixel was classified according to the direction of change in vegetation coverage and the corresponding climatic variable. Four interaction types were identified: climate-variable increase with vegetation increase, climate-variable increase with vegetation decrease, climate-variable decrease with vegetation increase, and climate-variable decrease with vegetation decrease. These classifications were used to identify areas where vegetation and climate changed synchronously or in opposite directions.

#### 2.4.4. Directional Interaction Classification

To further identify the coordinated and non-coordinated relationships between vegetation coverage and climatic variables, each pixel was classified according to the sign of the interannual trend in vegetation coverage and the sign of the corresponding climatic variable. Four interaction types were defined: variable increase with coverage increase, variable increase with coverage decrease, variable decrease with coverage increase, and variable decrease with coverage decrease. The first and fourth types were interpreted as coordinated changes when both variables changed in the same direction, whereas the second and third types were interpreted as non-coordinated changes when the two variables changed in opposite directions.

## 3. Results

### 3.1. Impact of Temperature on Vegetation Coverage

#### 3.1.1. Correlation Between Annual Mean Temperature and Vegetation Coverage

**Figure 2:**
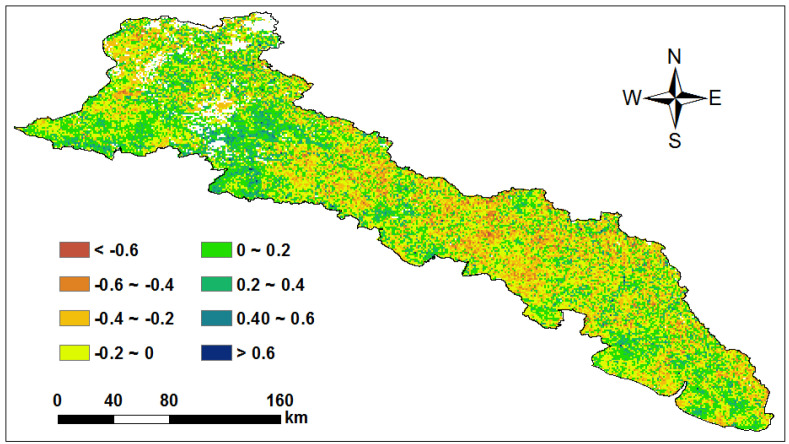
Spatial distribution of correlation coefficients between annual mean temperature and vegetation coverage (The white areas in the figure represent regions where no data were acquired during data collection).

The correlation between annual mean temperature and vegetation coverage showed strong spatial heterogeneity across the Chinese Altay Mountains. The area percentages for different correlation coefficient ranges are summarized in Table 2, and the spatial distribution is displayed in Figure 2.

As presented in Table 2 and Figure 2, negative correlations are more widespread across the study area, covering 57.61% of the total area, while positive correlations account for 42.39%. Within the negative correlation classes, the weak-to-moderate interval of −0.2 to 0 represents the largest share, reaching 32.90%, followed by the −0.4 to −0.2 class, which covers 19.56%. Areas with strong negative correlations below −0.4 are relatively limited, together occupying only 5.15% of the region, including 0.46% and 4.69% in the corresponding classes. For positive correlations, the 0 to 0.2 interval is dominant, accounting for 28.92% of the study area, whereas areas with stronger positive correlations above 0.2 represent only 13.47% in total, including 11.64%, 1.70%, and 0.13%. Overall, these results suggest that in most parts of the Altay Mountains, increases in annual mean temperature tend to be associated with reduced vegetation coverage, although this relationship is mainly weak to moderate. In terms of spatial distribution, negative correlations are mainly concentrated in the central and southeastern regions, while positive correlations occur as scattered patches, particularly in the higher-elevation areas of the northwest.

#### 3.1.2. Response of Vegetation Coverage to Temperature Changes

**Figure 3:**
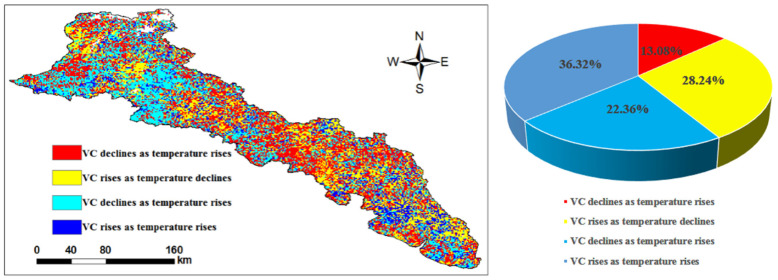
Spatial distribution of interactive relationships between temperature and vegetation coverage (VC) (The white areas in the figure represent regions where no data were acquired during data collection).

To further examine the response of vegetation coverage to interannual temperature variability, the study area was divided into four interaction types according to the direction of change in both temperature and vegetation coverage during 2000–2024. Specifically, the classification was based on whether the temperature trend and vegetation coverage trend were positive or negative: (i) warming with coverage increase, (ii) warming with coverage decrease, (iii) cooling with coverage increase, and (iv) cooling with coverage decrease. The area proportions of these four types are presented in Table 3, and their spatial distribution is illustrated in Figure 3.

As shown in Table 3 and Figure 3, the study area exhibits considerable spatial heterogeneity in the matching pattern between temperature change and vegetation response. Areas where vegetation coverage changes in the same direction as temperature, representing coordinated variation, account for 41.32% of the study area. This includes regions with simultaneous warming and vegetation coverage increase (13.08%) and those with concurrent cooling and vegetation coverage decline (28.24%). By contrast, uncoordinated patterns are more extensive, covering 58.68% of the region. These include areas where vegetation coverage increases under cooling conditions (22.36%) and areas where vegetation coverage decreases despite warming (36.32%).

Among all categories, the “warming and coverage decrease” type is the most widespread, occupying 36.32% of the total area. This result indicates that rising temperature generally exerts an adverse influence on vegetation growth in the Altay Mountains. The second largest category is “cooling and coverage decrease” (28.24%), suggesting that temperature decline and vegetation degradation occur simultaneously in many areas, possibly under the influence of additional constraints such as limited water availability. The smallest proportion is observed for the “warming and coverage increase” type (13.08%), implying that warming benefits vegetation only in restricted zones, probably in high-altitude areas where low temperature remains the dominant growth limitation.

Spatially, vegetation decline associated with warming is mainly distributed in the low and middle mountain belts, whereas vegetation improvement under cooling conditions occurs more frequently in the northwestern high-elevation areas. Overall, these findings demonstrate that the linkage between temperature variation and vegetation dynamics is spatially heterogeneous and complex. Nevertheless, warming appears to have an overall negative effect on vegetation coverage across much of the Chinese Altay Mountains.

### 3.2. Impact of Precipitation on Vegetation Coverage

#### 3.2.1. Areal Proportions of Correlation Coefficients

**Figure 4:**
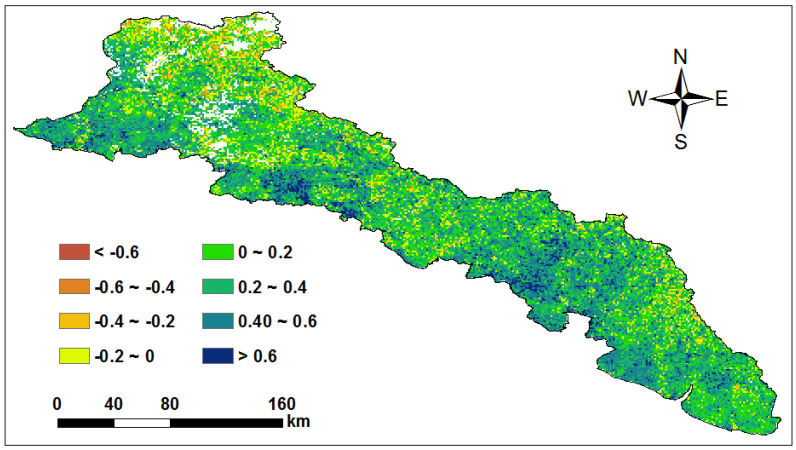
Spatial distribution of correlation coefficients between annual precipitation and vegetation coverage (The white areas in the figure represent regions where no data were acquired during data collection).

Annual precipitation showed a predominantly positive relationship with vegetation coverage across the study area. The resulting correlation coefficients (R) were classified into eight categories to reveal the spatial relationship between vegetation coverage and precipitation. The areal proportions for each category are presented in Table 4 and illustrated in Figure 4.

The results show that vegetation coverage is mainly positively correlated with annual precipitation throughout the study area. Areas with positive correlations (R > 0) account for 84.84% of the total region, whereas negative correlations (R < 0) are limited to 15.16%.

This broadly positive association emphasizes the importance of water supply in supporting vegetation growth in the Altay Mountains, where the continental climate is characterized by relatively limited annual precipitation, ranging from approximately 200 to 800 mm depending on elevation. Among the positive correlation classes, moderate positive correlations are the most widely distributed. The intervals of 0.2 < R ≤ 0.4 and 0.4 < R ≤ 0.6 account for 35.32% and 22.90% of the study area, respectively, together covering 58.22%. Weak positive correlations, with 0 < R ≤ 0.2, occupy 22.72% of the region, whereas strong positive correlations above 0.6 are relatively limited, covering only 3.90%.

For areas with negative correlations, weak negative relationships, ranging from −0.2 < R ≤ 0, are dominant and account for 10.32% of the total area. In contrast, stronger negative correlations with R ≤ −0.4 are rare, representing less than 1% of the study area, specifically 0.99%. Compared with the relationship between temperature and vegetation coverage, the precipitation–vegetation correlations are generally stronger and more consistently positive. This indicates that precipitation acts as a more direct and dominant constraint on vegetation productivity across much of the Altay Mountains, especially in the low and middle mountain belts where water limitation is more evident.

#### 3.2.2. Areal Proportions of Coordinated and Non-Coordinated Changes

**Figure 5:**
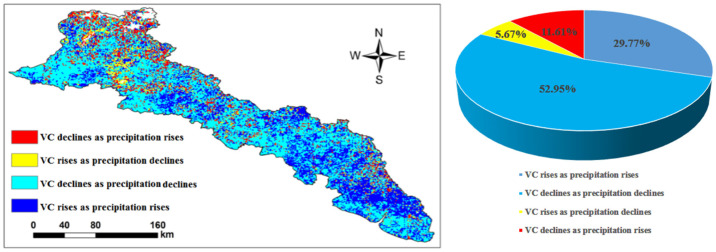
Spatial distribution of interactive relationships between precipitation and vegetation coverage (VC) (The white areas in the figure represent regions where no data were acquired during data collection).

To further clarify the interaction between precipitation variability and vegetation dynamics, interannual trends in vegetation coverage were directly compared with corresponding changes in precipitation. For this purpose, each pixel was assigned to one of four categories according to whether vegetation coverage and precipitation increased or decreased during the study period (Figure 5). This classification separates areas with coordinated changes, where both variables show the same trend direction, from areas with non-coordinated changes, where the two variables change in opposite directions.

The results indicate that coordinated variations are dominant across the study area, representing 82.72% of the total region. This finding is consistent with the correlation analysis and further confirms that vegetation coverage in most parts of the Altay Mountains responds positively to precipitation variability. Within the coordinated-change category, the most widespread pattern is “decreasing precipitation and declining vegetation coverage,” which accounts for 52.95% of the total area. This proportion is almost twice that of the “increasing precipitation and increasing vegetation coverage” pattern, which covers 29.77%. This asymmetry does not by itself demonstrate a regional drying trend; rather, it suggests that vegetation coverage was more frequently associated with precipitation decreases, or that vegetation was more sensitive to reductions in water supply than to precipitation gains. Non-coordinated changes cover a much smaller proportion of the study area, accounting for 17.28%. Among these patterns, the combination of increasing precipitation and declining vegetation coverage is more common, occupying 11.61% of the region. This is more than twice the area represented by decreasing precipitation and increasing vegetation coverage, which accounts for 5.67%.

The “precipitation increase and coverage decrease” pattern may occur where the beneficial effects of higher rainfall are weakened or offset by other limiting factors, such as enhanced evapotranspiration caused by rising temperature, or local disturbances including fire and grazing. In contrast, the relatively limited “precipitation decrease and coverage increase” pattern may be related to vegetation with deeper root systems, such as Siberian larch, which can access groundwater, or to favorable microclimatic conditions in high-elevation areas. Overall, although vegetation coverage generally shows a positive and coordinated response to precipitation change, the existence of non-coordinated patterns indicates marked spatial heterogeneity and the additional influence of other environmental controls.

### 3.3. Impact of Drought on Vegetation Coverage

#### 3.3.1. Areal Proportions of Correlation Coefficients with SPEI and TVDI

**Figure 6:**
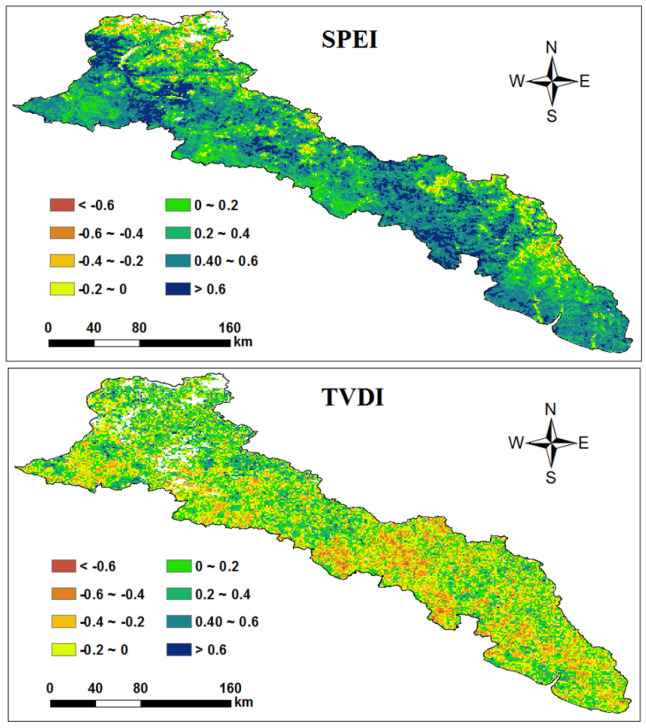
Spatial distribution of correlation coefficients between drought indicators and vegetation coverage (The white areas in the figure represent regions where no data were acquired during data collection).

To evaluate how drought affects vegetation dynamics in the Chinese Altay Mountains, pixel-level Pearson correlation analysis was conducted between annual maximum NDVI, used here to represent peak vegetation coverage, and two commonly applied drought indicators: the Standardized Precipitation–Evapotranspiration Index (SPEI) and the Temperature Vegetation Dryness Index (TVDI). SPEI characterizes meteorological drought by describing the climatic water balance, namely the difference between precipitation and potential evapotranspiration, whereas TVDI indicates surface soil moisture status based on the relationship between land surface temperature and NDVI. The area percentages of different correlation coefficient classes for the two drought indices are presented in Table 5 and shown in Figure 6.

For SPEI, vegetation coverage shows a distinctly positive association across most of the study area. Areas with positive correlations (R > 0) account for 89.71% of the region, while negative correlations are limited to 10.29%. Within the positive correlation classes, moderate and strong relationships occupy the largest proportions. Specifically, the 0.2–0.4 correlation class covers 28.72% of the area, the 0.4–0.6 class accounts for 34.04%, and strong positive correlations above 0.6 represent 11.61%. These results suggest that in most parts of the Altay Mountains, higher SPEI values, reflecting wetter climatic conditions, correspond to greater vegetation coverage, whereas lower SPEI values, indicating drier conditions, are generally linked to reduced vegetation coverage.

The spatial pattern shown in Figure 6 indicates that positive SPEI–vegetation correlations are especially stable in the middle-elevation forest and grassland zones. In these areas, vegetation growth is strongly constrained by water availability because of the continental climate and moderate annual precipitation, generally ranging from 300 to 500 mm.

By contrast, TVDI is mainly negatively correlated with vegetation coverage, which is consistent with its physical interpretation, because higher TVDI values reflect stronger surface dryness and greater plant water stress. The results show that negative correlations (R < 0) cover 54.25% of the study area, whereas positive correlations account for 45.75%. Most negative correlations fall within the weak to moderate ranges. Specifically, the −0.2 to 0 class accounts for 28.93% of the area, the −0.4 to −0.2 class covers 18.96%, and the −0.6 to −0.4 class represents 5.80%. Strong negative correlations below −0.6 are very limited, occupying only 0.56% of the region.

Positive correlations between TVDI and vegetation coverage, where higher surface dryness occurs together with greater vegetation coverage, are generally weaker. These areas are mainly distributed in the 0 to 0.2 class, covering 25.83%, and the 0.2 to 0.4 class, covering 14.73%. The contrasting correlation patterns of SPEI and TVDI correspond well to their different meanings. SPEI reflects the integrated climatic water balance and therefore tends to increase under wetter conditions favorable for vegetation growth. TVDI, however, represents short-term thermal and moisture stress at the land surface. The relatively weaker and more heterogeneous TVDI response may be associated with the mixed distribution of forests, wetlands, and grasslands, as well as the ability of some deep-rooted species, such as Siberian larch, to sustain transpiration under moderate surface drying.

#### 3.3.2. Areal Proportions of Coordinated and Non-Coordinated Changes with SPEI and TVDI

**Figure 7:**
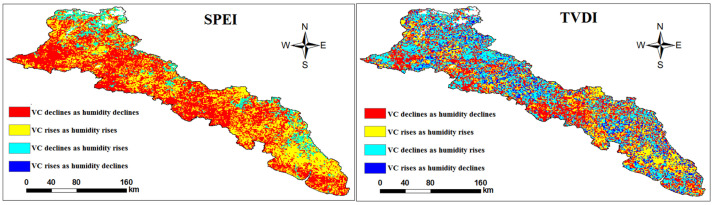
Spatial distribution of interactive relationships between drought indicators and vegetation coverage (VC) (The white areas in the figure represent regions where no data were acquired during data collection).

In addition to correlation analysis, the interannual directional changes between vegetation coverage and each drought index were further examined (Figure 7; Table 6). For this analysis, each pixel was grouped into one of four categories according to whether vegetation coverage and the corresponding drought indicator increased simultaneously, decreased simultaneously, or exhibited opposite trends. These categories represent coordinated positive change, coordinated negative change, and non-coordinated change patterns.

For SPEI, coordinated variations account for 89.71% of the study area, which is highly consistent with the proportion of positive correlations. Within this category, the pattern of decreasing SPEI accompanied by declining vegetation coverage is the most extensive, covering 54.27% of the total region. In comparison, the pattern of increasing SPEI together with increasing vegetation coverage accounts for 35.44%. This imbalance between the two coordinated types, 54.27% versus 35.44%, indicates that reduced water availability was more widespread during 2000–2024 and exerted a stronger negative influence on vegetation than wetter conditions which exerted a positive effect.

Non-coordinated changes occupy only 10.29% of the study area. This category is entirely represented by areas where SPEI increased but vegetation coverage decreased, whereas no area showed vegetation greening under decreasing SPEI conditions. The absence of the “drier but coverage increase” pattern suggests that no pixel exhibited a clear increase in vegetation coverage while meteorological drought intensified. This further supports the conclusion that meteorological drought is a major limiting factor controlling vegetation dynamics in the Altay Mountains.

For TVDI, the vegetation–drought interaction shows a more complicated pattern, reflecting the mixed correlation results. Coordinated changes, where TVDI and vegetation coverage vary in the expected direction, cover 55.70% of the study area. This includes areas where increasing surface dryness, indicated by rising TVDI, is accompanied by declining vegetation coverage (33.82%), as well as areas where decreasing TVDI, representing wetter surface conditions, occurs together with increasing vegetation coverage (21.88%). This proportion is slightly higher than the area showing negative TVDI–vegetation correlations (54.25%), suggesting that vegetation coverage generally decreases when surface dryness intensifies.

However, non-coordinated changes still occupy a large proportion of the region, accounting for 44.30%. These include areas where TVDI decreases but vegetation coverage also declines (30.74%) and areas where TVDI increases while vegetation coverage rises (13.56%). The relatively high proportion of the “wetter but coverage decreases” pattern is especially important, as it indicates that in nearly one-third of the Altay Mountains, reduced surface dryness or improved moisture conditions did not necessarily lead to increased vegetation coverage. This phenomenon may be related to several mechanisms. First, vegetation recovery may lag behind moisture improvement because plants require more than one growing season to recover from previous drought stress. Second, low temperature may still restrict plant growth in high-elevation zones even when soil moisture conditions are favorable. Third, local disturbances, such as grazing or fire, may weaken vegetation recovery. Fourth, TVDI mainly reflects moisture conditions in the surface soil layer, whereas deep-rooted forest species can obtain water from deeper soil or groundwater sources, thereby weakening the linkage between surface moisture variation and canopy response.

In contrast, the 13.56% of areas showing increasing TVDI together with increasing vegetation coverage may represent zones where drought-tolerant or non-native species expand under drier conditions, or where reduced moisture is accompanied by improved light or nutrient conditions. Overall, the comparison between SPEI and TVDI indicates that meteorological drought, represented by SPEI, has a more direct and spatially consistent negative influence on vegetation coverage in the Chinese Altay Mountains. By contrast, the vegetation response to surface soil dryness, represented by TVDI, is more spatially variable and is likely regulated by vegetation type, rooting depth, elevation-related temperature limitation, and local microclimatic conditions.

### 3.4. Impact of Extreme Climate Change on Vegetation Coverage

#### 3.4.1. Areal Proportions of Correlation Coefficients Between Vegetation Coverage and Extreme Climate Indices

**Figure 8:**
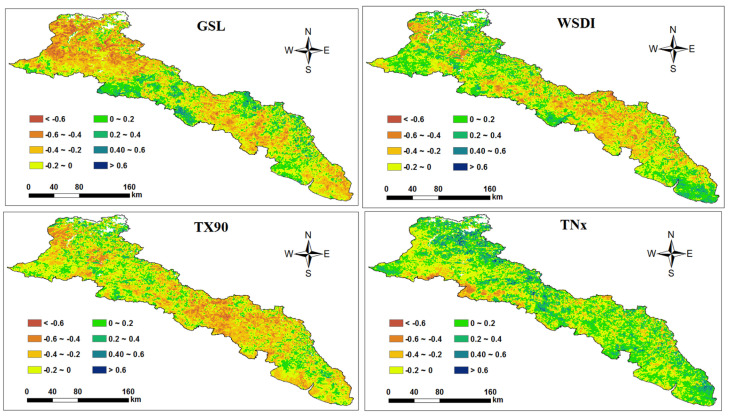

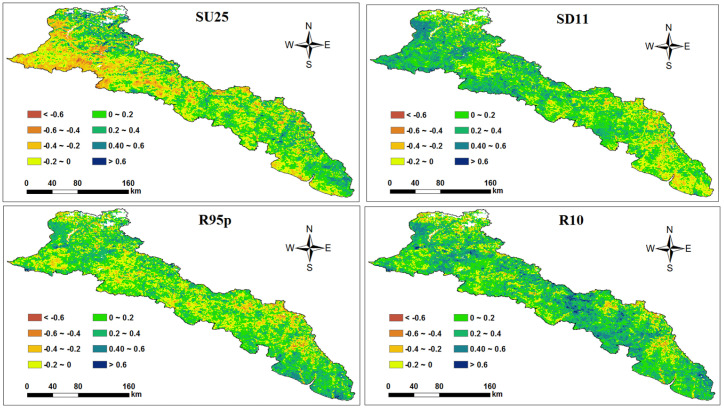
Spatial distribution of interactive relationships between extreme weather indicators and vegetation coverage (VC) The white areas in the figure represent regions where no data were acquired during data collection).

To assess the effects of extreme climate events on vegetation dynamics in the Chinese Altay Mountains, pixel-based Pearson correlation analyses were conducted between annual maximum NDVI, used as an indicator of peak vegetation coverage, and eight extreme climate indices for the period 2000–2024. These indices were divided into two categories: temperature-related extremes, including growing season length (GSL), warm spell duration index (WSDI), warm days (TX90), maximum daily minimum temperature (TNx), and summer days (SU25); and precipitation-related extremes, including precipitation intensity (SDII), heavy precipitation amount (R95p), and moderate rain days (R10). The area proportions of different correlation coefficient classes for these eight indices are summarized in Table 7 and displayed in Figure 8.

A distinct contrast is observed between temperature-related and precipitation-related extreme climate indices. For the temperature-extreme indicators GSL, WSDI, and TX90, vegetation coverage is mainly negatively correlated, suggesting that more frequent or intensified warm conditions generally suppress vegetation coverage. The areas showing negative correlations (R < 0) account for 75.00% for GSL, 64.29% for WSDI, and 78.08% for TX90.

Within the negative correlation classes, weak to moderate negative relationships account for the largest proportions. For GSL, the −0.4 to −0.2 and −0.2 to 0 classes occupy 29.61% and 31.90% of the study area, respectively. A similar pattern is observed for TX90, where these two classes cover 30.42% and 37.88%, respectively. This persistent negative association suggests that an extended growing season, more frequent consecutive warm days, and a higher occurrence of warm days may intensify vegetation water stress by increasing evapotranspiration. Such effects are particularly unfavorable in the Altay Mountains, where the continental climate and limited precipitation, especially in low and middle elevation zones, restrict water availability.

By contrast, TNx, representing the maximum daily minimum temperature, and SU25, representing summer days, show a more balanced spatial pattern. For TNx, positive correlations account for 53.95% of the area, while negative correlations cover 46.06%. For SU25, positive and negative correlations occupy 51.18% and 48.83%, respectively. This nearly even distribution indicates that warmer nighttime conditions and more summer days may promote vegetation growth in some areas, particularly at higher elevations where low temperature limits plant activity, but may inhibit vegetation in drier low mountain zones where additional warmth can aggravate moisture stress.

For the precipitation-related extreme indices, namely SDII, R95p, and R10, positive correlations are clearly dominant. Areas with positive correlations account for 74.99% for SDII, 67.85% for R95p, and 82.95% for R10. Most of these positive relationships are concentrated in the weak to moderate classes. For SDII, the 0 to 0.2 class covers 37.99% of the region, while the 0.2 to 0.4 class accounts for 28.81%. For R10, these two classes occupy 32.62% and 37.49%, respectively. Among the three precipitation indices, R10 shows the highest positive correlation area, reaching 82.95%, and also has the largest proportion of correlations greater than 0.4, with 12.84% in total across the 0.4 to 0.6 and >0.6 classes. This indicates that the frequency of moderate rainfall events is an especially important positive factor for vegetation coverage.

The positive response of vegetation to precipitation extremes is reasonable, as more frequent or stronger rainfall events can reduce soil moisture deficits and support plant growth in this water-limited mountain environment. However, differences among the precipitation indices are still evident. SDII and R95p show slightly weaker positive effects than R10, possibly because highly intense rainfall may generate more surface runoff and less effective infiltration, particularly on steep mountain slopes. Overall, the correlation results indicate that warmth-related extreme indices generally constrain vegetation coverage, whereas precipitation-related extremes tend to enhance it, although the strength and direction of these effects vary among indices and elevation zones.

#### 3.4.2. Areal Proportions of Coordinated and Non-Coordinated Changes Between Vegetation Coverage and Extreme Climate Indices

Although correlation coefficients describe the strength and direction of linear associations, the interaction analysis presented in Table 8 further evaluates how interannual changes in each extreme climate index correspond to changes in vegetation coverage. For this analysis, each pixel was assigned to one of four categories: (1) index increase accompanied by vegetation coverage increase, representing coordinated positive change; (2) index decrease accompanied by vegetation coverage decrease, representing coordinated negative change; (3) index decrease accompanied by vegetation coverage increase; and (4) index increase accompanied by vegetation coverage decrease. The latter two categories represent non-coordinated changes. Coordinated changes, defined as categories 1 and 2 combined, indicate a direct and synchronous response between vegetation coverage and the corresponding climate index, whereas non-coordinated changes suggest that vegetation responses are influenced or modified by additional environmental factors.

For the three temperature-related extreme indices that were mainly negatively correlated with vegetation coverage, namely GSL, WSDI, and TX90, the interaction analysis shows that opposite-direction changes are dominant. At first glance, this may appear inconsistent with the correlation results, but it actually reflects the negative response mechanism. Non-coordinated changes, defined as categories 3 and 4, account for 74.99% of the area for GSL, 64.29% for WSDI, and 78.08% for TX90. Within these categories, the most extensive pattern for both GSL and TX90 is “index increase with coverage decrease,” covering 49.81% and 50.57% of the study area, respectively. This means that in nearly half of the region, increases in growing season length or warm-day frequency occur together with reduced vegetation coverage, indicating that enhanced thermal extremes may intensify water stress and suppress vegetation growth.

A considerable proportion of the area also shows the opposite pattern, namely “index decrease with coverage increase,” accounting for 25.18% for GSL and 27.51% for TX90. This suggests that reduced warmth-related extremes, or relatively cooler conditions, are associated with vegetation improvement in many pixels. This pattern is also consistent with a negative relationship. Therefore, the relatively small proportions of coordinated changes, such as simultaneous decreases or simultaneous increases in both variables, do not contradict the correlation results. For example, for GSL, the “decrease–decrease” and “increase–increase” categories account for only 14.75% and 10.26%, respectively. Instead, the dominance of non-coordinated changes indicates that the negative correlations are mainly driven by opposite directional trends between warmth-related extremes and vegetation coverage.

For TNx and SU25, whose correlation patterns are more balanced, coordinated changes are slightly more widespread, accounting for 53.94% and 51.18% of the study area, respectively. For TNx, the largest category is “index decrease with coverage decrease,” which covers 36.98%, while “index increase with coverage increase” accounts for 16.96%. This indicates that in many areas, lower nighttime minimum temperatures may be linked to reduced vegetation coverage, possibly because of cold or frost limitation in high-elevation zones. Conversely, higher minimum temperatures may promote vegetation growth in areas where low temperature is a major constraint. A similar pattern is observed for SU25, with coordinated decrease and coordinated increase accounting for 34.37% and 16.81%, respectively.

For the three precipitation-related extreme indices, SDII, R95p, and R10, coordinated changes are dominant, which agrees with their generally positive correlations with vegetation coverage. Coordinated changes account for 74.98% of the area for SDII, 67.84% for R95p, and 82.95% for R10. Among these, the largest category is “index decrease with coverage decrease,” accounting for 46.74% for SDII, 39.72% for R95p, and 50.80% for R10. The “index increase with coverage increase” category is also substantial, covering 28.24%, 28.12%, and 32.15% for SDII, R95p, and R10, respectively.

The larger proportion of coordinated negative changes compared with coordinated positive changes suggests that, during 2000–2024, reductions in precipitation-related extremes, including fewer moderate rainfall days, lower precipitation intensity, and reduced heavy precipitation, were more widespread and had a stronger negative effect on vegetation coverage than increases in these indices had a positive effect. This is consistent with the asymmetric vegetation response observed for annual precipitation. Non-coordinated patterns for precipitation indices are relatively limited. The “index decrease with coverage increase” category ranges from 3.30% for R10 to 7.32% for R95p, whereas the “index increase with coverage decrease” category ranges from 13.76% for R10 to 24.84% for R95p. The relatively small area of “increase–decrease” patterns suggests that, unlike thermal extremes, stronger or more frequent precipitation extremes rarely reduce vegetation coverage in this water-limited mountain environment.

Overall, the interaction analysis further confirms that precipitation-related extremes generally support vegetation growth through coordinated changes, whereas warmth-related extremes exert a more complex but predominantly negative influence. In particular, increases in growing season length, warm spells, and warm-day frequency are often accompanied by vegetation decline, highlighting the importance of heat-induced water stress in shaping vegetation dynamics in the Chinese Altay Mountains.

## 4. Discussion

### 4.1. Water Availability as the Primary Limiting Factor

The dominant role of water availability observed in the Altay Mountains is consistent with evidence from several non-Asian regions. In Europe, recent drought events have caused widespread reductions in ecosystem greenness and forest canopy condition, demonstrating that vegetation responses to drought are often strongest where water balance becomes limiting [56]. A continental-scale European assessment also showed that excess forest mortality is strongly linked to drought and that mortality risk increases sharply once climatic water balance falls below a critical threshold [57]. Similarly, global analyses have shown that drought recovery times are closely related to climate and carbon-cycle dynamics, indicating that the effects of water deficit can persist beyond the drought year itself [58]. These findings support our interpretation that vegetation coverage in the Altay Mountains is primarily constrained by cumulative water availability rather than by precipitation amount alone.

The clearest and most consistent result of this study is that water availability plays a dominant positive role in regulating vegetation coverage across the Chinese Altay Mountains. This pattern is evident whether water conditions are represented by annual precipitation, the Standardized Precipitation–Evapotranspiration Index (SPEI), or precipitation-related extreme climate indices. More than 84% of the study area exhibited a positive correlation between vegetation coverage and annual precipitation, while areas with coordinated changes, where both variables increased or decreased together, covered nearly 83% of the region. These findings are consistent with the widely recognized view that in continental and water-limited environments, vegetation growth is mainly controlled by moisture supply rather than temperature alone [59,60].

Interestingly, the asymmetric pattern of coordinated changes—52.95% of the area experienced “precipitation decrease with coverage decrease”, while only 29.77% showed “precipitation increase with coverage increase”—suggests that during the study period (2000–2024), the larger area of “precipitation decrease with coverage decrease” indicates that vegetation decline was more frequently associated with reductions in precipitation than vegetation increase was associated with precipitation gains. However, because this directional interaction analysis does not constitute a formal trend analysis of annual precipitation, this result should be interpreted as an asymmetric vegetation response to lower water availability rather than direct evidence of a regional drying trend. This asymmetric response may be explained by two complementary mechanisms. First, the Altay Mountains already experience relatively dry baseline conditions, particularly in the low and middle elevation zones where annual precipitation is generally only 200–500 mm. Under such conditions, even a modest decline in rainfall may quickly push vegetation beyond critical physiological thresholds, leading to a marked reduction in vegetation coverage. Second, concurrent warming during the study period likely increased evapotranspiration demand, thereby intensifying the negative effects of precipitation shortage. Comparable asymmetric vegetation responses have also been observed in semi-arid regions of Inner Mongolia and Central Asia [61,62].

### 4.2. The Dual Role of Temperature: Cold Limitation at High Elevations vs. Heat Stress in Low Belts

In contrast to the consistently positive influence of precipitation, temperature showed a more complicated and elevation-dependent association with vegetation coverage. Negative correlations covered 57.6% of the study area, and the largest interaction type was “warming accompanied by vegetation coverage decline” (36.3%). These results suggest that increasing temperature has generally had an unfavorable effect on vegetation across much of the Altay Mountains. This response is most likely related to the enhancement of atmospheric evaporative demand under warmer conditions, which can accelerate soil moisture loss and intensify plant water stress. Such a mechanism has been widely reported in arid and semi-arid mountain environments [63,64].

Similar mechanisms have been reported in western North America, where drought and heat stress can interact with biotic disturbances to increase forest vulnerability and tree mortality [65]. This supports the interpretation that warming may benefit vegetation in cold-limited high-elevation zones, but may suppress vegetation in low- and middle-elevation belts where atmospheric water demand and soil moisture limitation are stronger.

Nevertheless, positive correlations, which covered 42.4% of the study area, together with the patterns of “warming with vegetation coverage increase” (13.1%) and “cooling with vegetation coverage decrease” (28.2%), suggest that temperature limitation remains important at higher elevations. In the northwestern high-altitude zones above 2000 m, low temperature is likely a major constraint on vegetation growth, especially for cold-tolerant coniferous species such as Siberian larch and spruce. In these areas, moderate warming may lengthen the growing season, improve photosynthetic activity, and support biomass accumulation. This contrasting role of temperature, with negative effects in water-limited lowlands but positive effects in cold-limited highlands, has also been reported in other mountain systems, including the Tianshan Mountains and the Tibetan Plateau [66,67]. The spatial patterns shown in Figure 2 and Figure 3 further support this elevation-dependent transition.

### 4.3. Divergent Responses to Meteorological Drought (SPEI) and Surface Dryness (TVDI)

The contrasting correlation patterns between SPEI and TVDI provide important insights into the multi-scale nature of drought impacts on vegetation in the Chinese Altay Mountains. SPEI, which integrates precipitation and potential evapotranspiration over a multi-month scale, showed an overwhelmingly positive relationship with vegetation coverage, with positive correlations covering 89.7% of the study area. This result indicates that cumulative climatic water balance, rather than precipitation alone, is a key determinant of vegetation condition in this mountain ecosystem. The absence of pixels showing “drier but coverage increase” in the SPEI interaction analysis further supports the conclusion that meteorological drought is a strong and consistent limiting factor for vegetation growth.

By contrast, TVDI, which is a remote-sensing indicator of surface soil moisture conditions, showed a weaker and more spatially variable relationship with vegetation coverage. Negative correlations accounted for only 54.3% of the study area, while non-coordinated changes covered 44.3%. The relatively high proportion of the “wetter conditions with declining vegetation coverage” pattern, indicated by decreasing TVDI but reduced vegetation coverage, is especially important, accounting for 30.7% of the region.

Several factors may explain this pattern. First, TVDI mainly represents moisture conditions in the near-surface soil layer, whereas dominant forest species such as Siberian larch often develop deep root systems that allow them to use deeper soil water or groundwater. This can weaken the linkage between canopy temperature and surface dryness. Second, TVDI is not controlled solely by soil moisture; it may also be affected by vegetation structure, terrain-induced shading, and atmospheric conditions. Third, vegetation recovery may lag behind improvements in surface moisture, meaning that delayed greening responses cannot be fully captured by a single-year comparison. These results are consistent with previous studies [53,64], which reported that TVDI is more effective in sparsely vegetated semi-arid landscapes but may be less reliable in dense forests and topographically complex mountain regions such as the Altay Mountains.

### 4.4. Extreme Climate Indices: Asymmetric Effects on Vegetation

The results for the eight extreme climate indices show a distinct contrast between temperature- and precipitation-related extremes. Warmth-related indices, including GSL, WSDI, and TX90, were mainly negatively correlated with vegetation coverage. Their interaction patterns were also dominated by “index increase with vegetation coverage decrease,” indicating that more frequent or prolonged warm conditions tend to suppress vegetation growth. This finding suggests that intensified warm extremes may increase evapotranspiration and aggravate vegetation water stress, especially during the growing season when plant water demand is high.

In contrast, TNx, representing the maximum daily minimum temperature, and SU25, representing summer days, showed a more balanced response. This suggests that warmer nighttime conditions may reduce frost limitation in high-elevation areas, while an increase in summer days may promote vegetation growth in cold-limited zones but intensify water stress in dry lowland areas. Such threshold-dependent vegetation responses have also been observed in boreal and alpine forest ecosystems [68,69].

Evidence from the high Andes also indicates that mountain vegetation responses are strongly shaped by topographic gradients and hydroclimatic variability, with vegetation productivity, surface water, and snow–ice indicators responding differently to temperature and water-balance conditions [70,71]. This comparison further supports the need to interpret vegetation–climate relationships in mountain systems as spatially heterogeneous and elevation-dependent.

For precipitation-related extremes, including SDII, R95p, and R10, the strong positive correlations and dominant coordinated changes indicate that more frequent or heavier rainfall events generally enhance vegetation growth. Among these indices, R10, representing moderate rain days, showed the highest proportion of positive correlations at 83.0%. This suggests that frequent moderate rainfall may be more effective in maintaining soil moisture than occasional heavy rainfall events represented by R95p. In mountainous terrain, intense rainfall can rapidly convert into surface runoff, especially on steep slopes, thereby reducing effective infiltration. These findings have important implications for future climate projections. If climate change increases rainfall intensity without increasing rainfall frequency, the positive effect on vegetation may be weaker than that produced by more frequent moderate rainfall events.

### 4.5. Limitations and Future Directions

Several limitations need to be considered. First, the study period from 2000 to 2024 is still relatively limited for identifying long-term climate trends, and the results may be affected by the particular years included in the analysis. Extending the time series, for example back to the 1980s, would help verify the observed asymmetric vegetation responses. Second, although the seven meteorological stations are relatively well distributed, they may not fully represent the strong spatial variability of temperature and precipitation in complex mountain terrain. Therefore, high-resolution gridded climate datasets or satellite-based climate products could be used in future studies to supplement station observations. Third, although the NDVI series was smoothed using the Savitzky–Golay filter, residual uncertainties caused by atmospheric interference, sensor-related errors, and data-processing procedures may still remain.

Fourth, non-climatic factors were not explicitly incorporated in this study. Human and natural disturbances, such as grazing pressure, fire events, forest harvesting, and land-use change, may partly explain the non-coordinated vegetation responses observed in some areas. Future studies should combine field-based biomass measurements, vertical soil moisture observations, and disturbance records to better separate the effects of climate variability from those of human activities. Finally, the delayed and legacy effects of drought and extreme climate events require further attention, because vegetation responses may extend over several years rather than occurring within a single year. Time-series methods, such as distributed lag models or wavelet analysis, could be useful for examining these lagged responses.

Despite these limitations, this study offers a spatially explicit and reliable assessment of vegetation–climate relationships in the Chinese Altay Mountains. The results emphasize the dominant control of water availability, the elevation-dependent influence of temperature, and the different effects of meteorological drought and surface dryness on vegetation dynamics. These findings provide useful scientific support for adaptive forest management and water resource planning in this climate-sensitive mountain region.

## 5. Conclusions

This study examined the spatiotemporal relationships between vegetation coverage and demonstrated that vegetation dynamics in the Chinese Altay Mountains are primarily regulated by vegetation–atmosphere water deficit. Water availability, reflected by precipitation, SPEI, and precipitation-related extreme indices, was the dominant positive control on vegetation coverage, whereas warming and warm extremes generally suppressed vegetation in water-limited low- and middle-elevation areas. In contrast, warming may benefit vegetation in high-elevation zones where low temperature remains a major growth constraint. The stronger and more consistent relationship between SPEI and vegetation coverage than TVDI further indicates that cumulative climatic water balance is more effective than surface dryness alone in explaining regional vegetation responses.

The main contribution of this study is that it moves beyond single-factor climate–vegetation analysis by integrating mean climate variables, drought indicators, extreme climate indices, and directional interaction classification. This framework helps identify not only where vegetation changes are synchronized with climate variability, but also where vegetation responses are decoupled by elevation, water limitation, rooting depth, or delayed recovery. These findings improve understanding of vegetation sensitivity in dryland mountain ecosystems and provide scientific support for ecological conservation, drought monitoring, and adaptive water-resource management in the Altay Mountains.

Future research should combine remote sensing with field observations of soil moisture profiles, groundwater availability, species composition, and physiological traits to clarify the mechanisms behind spatially heterogeneous vegetation responses. Process-based ecohydrological models and future climate scenarios are also needed to assess how continued warming, altered precipitation regimes, and more frequent extreme events may affect vegetation stability and ecosystem services in this climate-sensitive mountain region.

## Figures and Tables

**Table 1:** Definitions of extreme climate indicators used in this study, based on WMO/ETCCDI climate extreme index definitions and previous applications.

Types	Abbreviation	Extreme Climate Index	Unit	Definition
Relative index	TX90	Number of warm days	d	The number of days in a year when the daily maximum temperature is greater than the 90th percentile value of the reference period.
Absolute index	SU25	Summer days	d	The number of days during the year when the maximum daily temperature is greater than 25 °C
External index	TNx	Daily minimum temperature	°C	The maximum value of the daily minimum temperature of each month
Other index	WSDI	Consecutive warm days	d	The number of days with a daily maximum temperature greater than the 90th percentile value for 1961–2024, and for more than 6 consecutive days
	GSL	Biological growing season	d	The annual number of days between the first occurrence of at least six consecutive days with a daily mean temperature greater than 5 °C and the first occurrence after 1 July of at least six consecutive days with a daily mean temperature lower than 5 °C.
	R95p	Heavy precipitation	mm	The daily precipitation is greater than the total precipitation of the 95th percentile in the base period
	SDII	Precipitation intensity	mm/d	The ratio of total precipitation with daily precipitation ≥1.0 mm to the number of precipitation days
	R10 mm	Number of moderate rain days	d	The number of days with daily precipitation ≥10 mm

**Table 2:** Area proportions of correlation coefficients between annual mean temperature and vegetation coverage in the Altay Mountains.

	Range of Correlation Coefficients							
	<−0.6	−0.6~−0.4	−0.4~−0.2	−0.2~0	0~0.2	0.2~0.4	0.4~0.6	>0.6
Area proportion (%)	0.46	4.69	19.56	32.90	28.92	11.64	1.70	0.13

**Table 3:** Area proportions of interactive relationships between annual mean temperature and vegetation coverage in the Altay Mountains.

Interaction Type	Response Category	Area Proportion (%)
Warming with coverage increase	Coordinated change	13.08
Warming with coverage decrease	Non-coordinated change	36.32
Cooling with coverage increase	Non-coordinated change	22.36
Cooling with coverage decrease	Coordinated change	28.24
Total coordinated change	—	41.32
Total non-coordinated change	—	58.68

**Table 4:** Area proportions of correlation coefficients between annual mean precipitation and vegetation coverage in the Altay Mountains.

	Range of Correlation Coefficients							
	<−0.6	−0.6~−0.4	−0.4~−0.2	−0.2~0	0~0.2	0.2~0.4	0.4~0.6	>0.6
Area proportion (%)	0.11	0.88	3.85	10.32	22.72	35.32	22.90	3.90

**Table 5:** Area proportions of correlation coefficients between drought indicators and vegetation coverage in the Altay Mountains.

Drought Indicators	Range of Correlation Coefficients							
	<−0.6	−0.6~−0.4	−0.4~−0.2	−0.2~0	0~0.2	0.2~0.4	0.4~0.6	>0.6
SPEI	0.05	0.60	2.55	7.10	15.34	28.72	34.04	11.61
TVDI	0.56	5.80	18.96	28.93	25.83	14.73	4.57	0.62

**Table 6:** Spatial distribution of drought–vegetation interactions (drought indicators vs. VC).

Drought Indicators	Vegetation Coverage Increase as Humidity Increase	Vegetation Coverage Decrease as Humidity Decrease	Vegetation Coverage Increase as Humidity Decrease	Vegetation Coverage Decrease as Humidity Increase
SPEI (%)	35.44	54.27	0.00	10.29
TVDI (%)	21.88	33.82	13.56	30.74

**Table 7:** Area proportions of correlation coefficients between extreme weather indicators and vegetation coverage in the Altay Mountains.

Extreme Weather Indicators	Range of Correlation Coefficients							
	<−0.6	−0.6~−0.4	−0.4~−0.2	−0.2~0	0~0.2	0.2~0.4	0.4~0.6	>0.6
GSL	1.66	11.83	29.61	31.90	17.85	6.03	1.08	0.04
WSDI	0.31	6.42	22.92	34.64	26.80	7.97	0.88	0.05
TX90	0.45	9.33	30.42	37.88	17.69	3.70	0.52	0.01
TNx	0.08	1.64	8.92	35.42	36.45	14.20	3.04	0.26
SU25	0.16	3.31	16.02	29.34	30.02	16.22	4.37	0.57
SDII	0.02	0.43	4.13	20.43	37.99	28.81	8.03	0.16
R95p	0.06	0.90	6.42	24.77	38.64	24.82	4.22	0.17
R10	0.03	0.61	3.61	12.81	32.62	37.49	11.98	0.86

**Table 8:** Areal proportions of extreme weather–vegetation correlation coefficients in the Altay Mountains.

Extreme Weather Indicators	Vegetation Coverage Increase as Indicator Increase	Vegetation Coverage Decrease as Indicator Decrease	Vegetation Coverage Increase as Indicator Decrease	Vegetation Coverage Decrease as Indicator Increase
GSL	10.26	14.75	25.18	49.81
WSDI	8.57	27.13	26.87	37.42
TX90	7.93	13.99	27.51	50.57
TNx	16.96	36.98	18.48	27.58
SU25	16.81	34.37	18.63	30.19
SDII	28.24	46.74	7.20	17.82
R95p	28.12	39.72	7.32	24.84
R10	32.15	50.80	3.30	13.76

## Data Availability

Data could be provided on reasonable request from the first author.
